# Lipoprotein(a) as an Independent Biomarker of Coronary Complexity: Prevalence, Clinical Correlates, and Diagnostic Utility in a North Indian Acute Coronary Syndrome Cohort

**DOI:** 10.7759/cureus.90747

**Published:** 2025-08-22

**Authors:** Kunal Mahajan, Surender Himral, Jai Bharat Sharma, Shivali Sandal, Tanuj Bhatia, Roshan Thakur, Iva Patel, Komal Mohite, Pankaj Chandel, Jaikrit Bhutani

**Affiliations:** 1 Cardiology, Himachal Heart Institute, Mandi, IND; 2 Critical Care Medicine, Himachal Heart Institute, Mandi, IND; 3 Cardiology, Shri Mahant Indiresh Hospital, Dehradun, IND; 4 Internal Medicine, Himachal Heart Institute, Mandi, IND; 5 Cardiology, Holy Heart Hospital, Rohtak, IND

**Keywords:** : acute coronary syndrome, coronary angiography, coronary artery disease, lipoprotein (a), syntax score

## Abstract

Background

Despite extensive research on coronary artery disease (CAD), many underlying reasons remain unexplored. Geographic variations warrant focused studies tailored to specific populations. This study was conducted to assess the role of lipoprotein(a) (Lp(a)) in complex CAD among the North Indian cohort with acute coronary syndrome (ACS).

Methods

This retrospective study included 688 ACS patients confirmed by ECG, cardiac biomarkers, and coronary angiography. They were grouped by Lp(a) levels (≤50 mg/dL and >50 mg/dL) to assess their association with SYNTAX score >22. Multivariate logistic regression evaluated the odds ratio of Lp(a) > 50 mg/dL, and sensitivity and specificity were analysed for different Lp(a) cut-off values.

Results

In the present study, 207 (30.1%) of participants had elevated Lp(a) levels exceeding 50 mg/dL. Comorbidities and baseline blood parameters were evenly distributed between groups. A SYNTAX score > 22 was observed in 35.7% (n = 74/207) of individuals with Lp(a) > 50 mg/dL, compared to 26.6% (n = 128/481) in those with Lp(a) ≤ 50 mg/dL. On univariate logistic regression, elevated Lp(a) was significantly associated with a high SYNTAX score (OR=1.53; 95% CI:1.08-2.17). This association remained statistically significant after adjustment for traditional cardiovascular risk factors (OR = 1.54; 95% CI: 1.07-2.21; p = 0.02). Sensitivity and specificity analysis identified the >50 mg/dL threshold as the optimal cut-off, yielding a sensitivity and specificity of 63.37% and 75.14%.

Conclusion

Lp(a) > 50 mg/dL was significantly associated with complex CAD, independent of traditional cardiovascular risk. This cut-off demonstrated good diagnostic performance, with a sensitivity of 63.37%, specificity of 75.14%, and overall accuracy of 71.91%, supporting its role as an independent biomarker for anatomical CAD complexity.

## Introduction

Lipoprotein(a), or Lp(a), is recognised as a significant genetically inherited and independent risk factor for cardiovascular disease (CVD). Its plasma concentration is largely determined by genetic factors and remains relatively stable throughout an individual’s life. Elevated levels of Lp(a) contribute to the development of atherosclerotic cardiovascular disease (ASCVD) by promoting inflammation and thrombogenesis. It plays a critical role in the initiation and progression of atherosclerosis and is commonly detected in advanced plaques and at sites of plaque rupture. While elevated low-density lipoprotein cholesterol (LDL-C) is a well-established driver of atherosclerosis, growing evidence indicates that other atherogenic lipoproteins, such as Lp(a), may also have a direct causal role in the disease process [1-3].

Globally, CVDs - including ischemic heart disease and stroke - remain the leading cause of mortality, accounting for an estimated 17.7 million deaths annually. According to the World Health Organization, India is responsible for nearly one-fifth of these global deaths, with a disproportionate impact observed among younger individuals. The Global Burden of Disease study reports an age-standardised CVD mortality rate of 272 per 100,000 in India, notably exceeding the global average of 235 per 100,000 [4].

In India, approximately 25% of the population is estimated to have elevated Lp(a) levels, indicating that one in four individuals may have concentrations above the threshold associated with increased cardiovascular risk. Despite significant advancements in diagnostics and treatment, the rate of sudden cardiac deaths continues to rise both in India and worldwide [5].

This ongoing public health concern highlights the importance of investigating cardiovascular risk factors more comprehensively, especially in relation to genetic background, regional diversity, lifestyle patterns, and dietary habits. These factors may influence the development and progression of CVD in distinct ways across populations. Although extensive research has been conducted on traditional risk factors, critical gaps remain - particularly concerning the role of Lp(a) - necessitating further in-depth and population-specific studies.

The present study aims to evaluate Lp(a) levels and examine their association with the complexity of coronary artery disease (CAD) in Indian patients presenting with acute coronary syndrome (ACS), with the goal of contributing to a deeper understanding of Lp(a) as a potential biomarker and therapeutic target in cardiovascular risk management.

## Materials and methods

Study design and population

This retrospective observational study was conducted at our cardiac institute between February 2023 and June 2025. Ethical clearance was obtained from the Institutional Ethics Committee. Since it was a retrospective analysis, consent was waived.

The study population comprised all the patients who presented with an ACS, confirmed through electrocardiography (ECG), cardiac biomarker elevation and coronary angiography. Patients in whom Lp(a) levels were taken at the time of admission were considered for this analysis. Patients were excluded from the study if they had severe comorbid conditions such as active malignancy, end-stage renal disease, or significant hepatic dysfunction. Those with recent acute infections or a history of major cardiovascular surgeries or interventions, such as prior coronary artery bypass grafting or percutaneous coronary intervention, were also excluded. Patients with missing data or those with Lp(a) estimation done at some other hospitals were also excluded. Detailed information on demographic characteristics, clinical history, laboratory parameters and angiographic findings was collected for each participant from the hospital records.

Hypertension was defined as a recorded blood pressure greater than 140/90 mmHg or the current use of antihypertensive medication. Diabetes mellitus was identified based on an HbA1c value of 6.5% or higher, a random blood glucose level exceeding 200 mg/dL, or the use of antidiabetic medications. Smoking status was recorded as a current smoker if the individual was actively smoking at the time of assessment, or as an ex-smoker if the individual had previously smoked but had quit for at least six months.

To evaluate the severity and complexity of CAD, the SYNTAX score was calculated for each participant using the official online SYNTAX score calculator (https://syntaxscore.org/calculator/syntaxscore/frameset.htm). Based on their scores, patients were divided into two groups: those with a SYNTAX score of 22 or below, and those with a score greater than 22.

Blood investigation

In our centre, blood samples are usually collected from each ACS patient upon admission. The samples are analysed in the hospital laboratory, which is accredited by the National Accreditation Board for Testing and Calibration Laboratories (NABL). In the present analysis, we retrospectively collected reports of HbA1c, lipid profile, Lp(a), apolipoproteins A and B, blood urea, uric acid, creatinine, and serum glutamic-pyruvic transaminase (SGPT) (alanine aminotransferase (ALT)). The Lp(a) concentration was measured using the immunoturbidimetry method, following the manufacturer’s guidelines.

Statistical analysis

For statistical analysis, SPSS software (IBM SPSS Statistics for Windows, IBM Corp., Armonk, NY) was used. Patients were categorised into two groups based on their Lp(a) levels: those with concentrations ≤50 mg/dL and those with >50 mg/dL. This cut-off was chosen in accordance with existing evidence indicating that Lp(a) levels above 50 mg/dL are associated with an increased risk of CVD [6]. Categorical variables were presented as numbers and percentages, and comparisons were made using the chi-square test. Continuous variables were expressed as mean ± standard deviation and analysed using the independent Student's t-test. To identify the independent association between elevated Lp(a) levels and a SYNTAX score greater than 22, multivariate logistic regression analysis was performed, with adjustments for age, hypertension, diabetes, prior statin use, and smoking. Sensitivity, specificity, and diagnostic accuracy of various Lp(a) cut-off values were assessed using standard diagnostic test evaluation methods. A two-tailed p-value of less than 0.05 was considered statistically significant.

## Results

A total of 688 patients were included in the final analysis. Participants had a mean age of 61.83 ± 11.46 years, of whom 79.7% were male. Comorbidities included hypertension (n = 305, 44.3%) and diabetes (n = 196, 28.5%), while 37.9% (n = 261) were current smokers and 23.7% (n = 163) were ex-smokers. ST elevation myocardial infarction (STEMI) was the predominant ACS presentation (n = 478, 69.5%), followed by non-ST-elevation myocardial infarction (NSTEMI) (n = 139, 20.2%) and unstable angina (n = 71, 10.3%) (Table 1).

**Table 1:** Baseline characteristics of the study population

Variables	N = 688 N (%)
Age	61.83 ± 11.46
Gender	
Male	548 (79.7%)
Female	140 (20.3%)
Comorbidities	
Diabetes	196 (28.5%)
Hypertension	305 (44.3%)
Current smoker	261 (37.9%)
Ex-smoker	163 (23.7%)
Medical history	
Past history of acute coronary syndrome	52 (7.6%)
Past history of peripheral artery disease	09 (1.3%)
Past stroke history	18 (2.6%)
Family history	33 (4.8%)
Rehospitalization	88 (12.8%)
Type of acute coronary syndrome	
Unstable angina	71 (10.3%)
Non-ST-elevation myocardial infarction	139 (20.2%)
ST elevation myocardial infarction	478 (69.5%)

Single-vessel, double-vessel, and triple-vessel disease were observed in 29.5% (n = 203), 33.7% (n = 232), and 36.8% (n = 253) of cases, respectively (Table 2). The left anterior descending (LAD) artery was the most frequently involved vessel (n = 368, 53.5%), followed by the right coronary artery (RCA) in 285 (41.4%) and the left circumflex (LCX) artery in 166 (24.1%) patients. Less common involvements included the obtuse marginal (OM) artery (n = 31, 4.5%), ramus (n = 5, 0.7%), and left main coronary artery (LMCA) (n = 39, 5.7%). The average SYNTAX score across the population was 17.19 ± 9.36.

**Table 2:** Angiographic parameters among study population

Variables	N = 688 N (%)
No. of vessels blocked	
Single vessel disease	203 (29.5%)
Double vessel disease	232 (33.7%)
Triple vessel disease	253 (36.8%)
Type of vessels involved	
Left anterior descending artery	368 (53.49%)
Right coronary artery	285 (41.42%)
Left circumflex coronary artery	166 (24.13%)
Obtuse marginal artery	31 (4.50%)
Ramus intermedius artery	05 (0.73%)
Left main coronary artery	39 (5.7%)
SYNTAX score	17.19 ± 9.36

The comparison between patients with Lp(a) levels of ≤50 mg/dL and >50 mg/dL showed no significant differences in age, gender, diabetes, hypertension, smoking status, type of ACS, prior cardiovascular events, BMI, or ejection fraction (Table 3). Additionally, no significant variation was observed across ejection fraction categories between the two groups.

**Table 3:** Comparison of baseline parameters among patients with lipoprotein(a) ≤ 50 and lipoprotein(a) > 50 ACS = acute coronary syndrome; EF = ejection fraction; MI = myocardial infarction; NSTEMI = non-ST-elevation myocardial infarction; STEMI = ST elevation myocardial infarction ^#^Chi-square test was used to compare the groups. *Student t-test was used to compare the means.

Variables	Lipoprotein(a) ≤ 50 (N = 481)	Lipoprotein(a) > 50 (N = 207)	p-value
Age in years (mean ± SD)	61.30 ± 11.57	63.04 ± 11.13	0.069^*^
Male	383 (79.6%)	165 (79.7%)	1.000^#^
Female	98 (20.4%)	42 (20.3%)	
Diabetes mellitus	138 (28.7%)	58 (28%)	0.927^#^
Hypertension	202 (42%)	103 (49.8%)	0.07^#^
Current smoker	189 (39.3%)	72 (34.8%)	0.149^#^
Ex-smoker	104 (21.6%)	59 (28.5%)	0.147^#^
USA	45 (9.4%)	26 (12.6%)	0.245^#^
NSTEMI	93 (19.3%)	46 (22.2%)	0.252^#^
STEMI	343 (71.3%)	135 (65.2%)	0.09^#^
Prior stain	89 (18.50%)	57 (27.53%)	0.01^#^
Past MI/ACS	36 (7.5%)	16 (7.7%)	0.876^#^
Past PAD history	06 (1.2%)	03 (1.4%)	1.000^#^
Past stroke history	11 (2.3%)	07 (3.4%)	0.438^#^
Family history	24 (5%)	09 (4.3%)	0.847^#^
BMI %	24.62 ± 5.57	24.27 ± 3.96	0.426^#^
EF % (mean ± SD)	40.93 ± 9.96	40.39 ± 10.46	0.521^*^
>50	82 (17.05%)	39 (18.84%)	0.6474^#^
41-49	108 (22.45%)	50 (24.15%)	0.6958^#^
<40	291 (60.50%)	118 (57%)	0.4404^#^

The comparison between patients with Lp(a) levels ≤50 mg/dL and >50 mg/dL showed no significant differences in age, gender, diabetes, hypertension, smoking status, type of ACS, prior cardiovascular events, BMI, or ejection fraction. Additionally, no significant variation was observed across ejection fraction categories between the two groups (Table 4).

**Table 4:** Comparison of blood investigation between the two lipoprotein(a) groups *Student t-test was used to compare the means between the two groups.

Variables	Lipoprotein(a) ≤ 50 (N = 481)	Lipoprotein(a) > 50 (N = 207)	p-value^*^
HbA1c %	6.22 ± 1.68	6.32 ± 1.62	0.491
Total cholesterol	174.73 ± 46.92	170.82 ± 53.41	0.337
High-density lipoprotein (HDL)	42.36 ± 11.69	42.28 ± 11.14	0.362
Low-density lipoprotein (LDL)	104.87 ± 34.67	101.42 ± 36.09	0.237
Very low-density lipoprotein (VLDL)	32.88 ± 22.99	29.42 ± 18.94	0.060
High-sensitivity C-reactive protein (mg/dL)	9.85 ± 19.79	10.42 ± 24.15	0.761
Apolipoprotein B (mg/dL)	83.13 ± 45.47	84.57 ± 26.67	0.671
Apolipoprotein A (mg/dL)	104.06 ± 20.45	102.99 ± 18.17	0.517
Blood urea (mg/dL)	15.28 ± 7.95	15.53 ± 7.53	0.706
Uric acid (mg/dL)	6.03 ± 1.79	6.22 ± 1.83	0.225
Creatinine (mg/dL)	1.4 ± 5.67	1.14 ± 0.44	0.533
Serum glutamic-oxaloacetic transaminase (SGOT) level (U/L)	115.29 ± 245.95	94.69 ± 109.43	0.420
Serum glutamic-pyruvic transaminase level (SGPT) (U/L)	72.59 ± 194.94	51.86 ± 40.65	0.293

Biochemical parameters showed no significant differences between the two groups for HbA1c, total cholesterol, high-density lipoprotein (HDL), low-density lipoprotein (LDL), very low-density lipoprotein (VLDL), high-sensitivity C-reactive protein (hs-CRP), apolipoproteins (A and B), blood urea, uric acid, creatinine, serum glutamic-oxaloacetic transaminase (SGOT), or SGPT. As expected, mean Lp(a) levels were markedly higher in the >50 mg/dL group (Table 5). In the angiographic analysis, multivessel disease was more frequently observed in patients with Lp(a) > 50 mg/dL, though the difference was not statistically significant. No significant differences were noted between the groups regarding the types of vessels involved, presence of left main CAD or mean SYNTAX score (Table 5). However, a significantly higher proportion of patients in the Lp(a) > 50 mg/dL group had a SYNTAX score > 22 (35.7% vs. 26.6%, p = 0.02), suggesting a greater extent of complex CAD (Table 6).

**Table 5:** Comparison of angiographic parameters among patients with lipoprotein(a) ≤ 50 and lipoprotein(a) > 50 ^#^Chi-square test was used to compare the two groups.

Variables	Lipoprotein(a) ≤ 50 (N = 481)	Lipoprotein(a) > 50 (N = 207)	p-value ^#^
No. of vessels blocked			
Single vessel disease (SVD)	150 (31.2%)	53 (25.6%)	0.1673
Multivessel disease (MVD)	331 (68.81%)	154 (74.40%)	
Type of vessels blocked			
Left anterior descending (LAD)	263 (54.7%)	105 (50.7%)	0.360
Right coronary artery (RCA)	199 (41.4%)	86 (41.5%)	1.000
Left circumference (LCX)	107 (22.2%)	59 (28.5%)	0.08
Obtuse marginal (OM)	25 (5.2%)	06 (2.9%)	0.231
Ramus intermedius	04 (0.8%)	01 (0.5%)	1.000
Left main coronary artery disease (LMCA)	23 (4.8%)	16 (7.74%)	0.150
SYNTAX score	16.86 ± 8.99	17.96 ± 10.16	0.170
≤22	353 (73.4%)	133 (64.3%)	0.02
>22	128 (26.6%)	74 (35.7%)	

**Table 6:** Association between elevated lipoprotein(a) levels (>50 mg/dL) and high coronary complexity (SYNTAX score > 22)

Variables		OR	95% CI	p-value
Lipoprotein > 50	Unadjusted	1.53	1.08-2.17	0.01
	Adjusted			
	Already on statin	1.63	1.13-2.33	0.01
	Age	1.48	1.04-1.111	0.03
	Hypertension	1.52	1.07-2.16	0.02
	Diabetes	1.56	1.10-2.22	0.01
	Smoking	1.56	1.09-2.21	0.01
	Age, hypertension, diabetes and smoking	1.54	1.08-2.21	0.02

Elevated Lp(a) levels (>50 mg/dL) were significantly associated with higher coronary complexity, defined as a SYNTAX score >22. In unadjusted analysis, the odds ratio (OR) was 1.53 (95% CI: 1.08-2.17; p = 0.01). After adjusting for statin use, the association remained significant (OR: 1.63; 95% CI: 1.13-2.33; p = 0.01). Further adjustments for age, hypertension, diabetes, prior statin use, and smoking continued to show a significant association (OR: 1.54; 95% CI: 1.08-2.21; p = 0.02), indicating that elevated Lp(a) is an independent predictor of more complex CAD (Table 6).

The diagnostic performance of various Lp(a) cut-offs was assessed for predicting a SYNTAX score >22. The >50 mg/dL threshold demonstrated the best overall performance, with a sensitivity of 63.37%, specificity of 75.14%, and the highest accuracy of 71.91% (Table 7). The corresponding positive and negative likelihood ratios were 2.55 and 0.49, respectively. Lower thresholds (e.g., >30 or >40 mg/dL) showed reduced accuracy, while higher cut-offs (>75 and >100 mg/dL) increased sensitivity but markedly decreased specificity and overall accuracy. These findings suggest that 50 mg/dL is the optimal Lp(a) cut-off for identifying complex CAD in this population.

**Table 7:** Diagnostic performance of lipoprotein(a) cut-off values in predicting SYNTAX score > 22

Statistic	Lipoprotein(a) > 30 (mg/dL)	Lipoprotein(a) > 40 (mg/dL)	Lipoprotein(a) > 50 (mg/dL)	Lipoprotein(a) > 75 (mg/dL)	Lipoprotein(a) > 100 (mg/dL)
Sensitivity	47.52%	57.92%	63.37%	78.22%	88.12%
Specificity	47.12%	35.19%	75.14%	17.28%	9.26%
Positive likelihood ratio	0.90	0.89	2.55	0.95	0.97
Negative likelihood ratio	1.11	1.20	0.49	1.26	1.28
Disease prevalence	29.36%	29.36%	27.41%	29.36%	29.36%
Positive predictive value	27.20%	27.08%	49.04%	28.21%	28.76%
Negative predictive value	68.36%	66.80%	84.45%	65.62%	65.22%
Accuracy	47.24%	41.86%	71.91%	35.17%	32.41%

## Discussion

In our study population comprising all-comers with ACS, we evaluated Lp(a) as a potential biomarker for assessing the burden of complex CAD. Elevated Lp(a) levels (>50 mg/dL) were observed in 207 (30.1%) individuals, indicating a substantial prevalence of high Lp(a) in this cohort. Importantly, a significantly greater proportion of patients in the Lp(a) >50 mg/dL group had a SYNTAX score >22 compared to those with lower levels (35.7% vs. 26.6%, p = 0.02), supporting an association between elevated Lp(a) and anatomically complex CAD. In multivariate logistic regression analysis, Lp(a) >50 mg/dL emerged as an independent predictor of a SYNTAX score >22, with an adjusted OR of 1.54 (95% CI: 1.08-2.21; p = 0.02). These findings suggest that even in individuals with otherwise normal lipid profiles, an elevated Lp(a) level is associated with an increased risk of complex CAD. Among the various cut-off thresholds tested, Lp(a) >50 mg/dL provided the best diagnostic performance, with a sensitivity of 63.37%, specificity of 75.14%, and the highest overall accuracy of 71.91%. The corresponding positive likelihood ratio was 2.55, indicating moderate diagnostic utility. Collectively, these results identify Lp(a) >50 mg/dL as the most appropriate and clinically meaningful threshold for predicting a SYNTAX score >22 in patients with ACS.

Previous studies have demonstrated a significant association between elevated Lp(a) levels and the severity of CAD as quantified by the SYNTAX score [7,8]. Kozieł-Siołkowska et al. [7] investigated patients with acute myocardial infarction (AMI) and reported a significant correlation between Lp(a) levels and a SYNTAX score ≥23. Their analysis identified a threshold of 166.16 nmol/L for Lp(a), which predicted a SYNTAX score ≥23 with a sensitivity of 97% and specificity of 44%. Similarly, Xu et al [8]. Evaluated patients with stable CAD and found that in individuals with LDL-C ≥ 100 mg/dL, an Lp(a) cut-off value >30 mg/dL was significantly associated with a SYNTAX score ≥23. In the present study, we found a sensitivity of 63.37% and a specificity of 75.14% for Lp(a) for predicting SYNTAX score > 22. Despite differences in the identified cut-off values, likely attributable to variations in study populations, comorbid conditions, and geographical factors, all studies consistently support the association between elevated Lp(a) levels and increased anatomical complexity of CAD, as reflected by higher SYNTAX scores.

Koziel et al. [7] further identified Lp(a) as an independent predictor of a SYNTAX score ≥23, reporting an OR of 1.03. In another study, Patel et al. [9] demonstrated that each 50 nmol/L increase in Lp(a) was associated with a hazard ratio(HR) of 1.11 for ASCVD, indicating a linear relationship between Lp(a) concentration and ASCVD risk. Notably, they observed significant interethnic differences in median Lp(a) levels, with values of 19, 31, 75, and 16 nmol/L among White, South Asian, Black, and Chinese populations, respectively. However, the associated risk per 50 nmol/L increase remained relatively consistent, with HRs of 1.11, 1.10, and 1.07 for White, South Asian, and Black individuals, respectively. These findings suggest that while baseline Lp(a) concentrations vary across racial groups, the relative cardiovascular risk conferred by elevated Lp(a) is comparable [9]. In the current study conducted among a North Indian cohort, an Lp(a) level exceeding 50 mg/dL was found to be associated with a SYNTAX score >22, supporting the link between elevated Lp(a) and more complex CAD. Collectively, these studies underscore both the variability of Lp(a) concentrations among different ethnic populations and the consistent association of elevated Lp(a) with increased cardiovascular risk and anatomical disease complexity. In the present study, we found the OR of Lp(a) > 50 to be 1.54 after adjusting for age, hypertension, diabetes and smoking.

Lp(a) is a genetically determined, monogenic risk factor for CVD, with 70-90% of level variability attributed to inherited factors. Strong genetic and epidemiological evidence links elevated Lp(a) with ASCVD and calcific aortic stenosis [10]. Despite its clinical relevance, routine testing is underutilised, and management protocols are lacking, contributing to a gap in both primary and secondary CVD prevention. Elevated Lp(a) levels (≥50 mg/dL or ≥125 nmol/L) affect over 1.5 billion people globally, reinforcing the need for universal screening and guideline implementation [11].

Several previous studies have underscored the significance of Lp(a) in the development and progression of CAD. In a cross-sectional study of 1,980 statin-naïve individuals undergoing coronary angiography, Sun et al. [12] identified Lp(a) as a useful biomarker for evaluating both the presence and burden of CAD. Similarly, Zhang et al. [13] found that elevated Lp(a) levels were independently linked to the presence and severity of CAD in patients with type 2 diabetes mellitus, highlighting its relevance in high-risk populations. The recent role of lipoprotein(a) in cardiovascular diseases and premature acute coronary syndromes (RELACS) study found that elevated Lp(a) is linked to earlier onset and greater complexity of CAD in ACS patients, emphasizing the need for targeted management strategies in individuals with high Lp(a), particularly in primary prevention [14].

There is currently no universal consensus on the threshold levels of Lp(a) associated with increased cardiovascular risk, as various international guidelines propose different cut-off values. The American College of Cardiology/American Heart Association (ACC/AHA) recognises≥50 mg/dL (≥125 nmol/L) as a risk-enhancing level [15]. Similarly, the Canadian Cardiovascular Society (CCS) recommends a threshold of ≥50 mg/dL (≥100 nmol/L) [15]. According to the European Atherosclerosis Society (EAS), Lp(a) levels are categorized as follows: <30 mg/dL (<75 nmol/L) is considered normal, 30-50 mg/dL (50-125 nmol/L) as intermediate, and >50 mg/dL (>125 nmol/L) as elevated or abnormal [15]. The National Lipid Association (NLA) also identifies >50 mg/dL (>100 nmol/L) as a level associated with increased cardiovascular risk [15]. The findings of our study are consistent with the Lp(a) thresholds recommended by the ACC/AHA and CCS, reinforcing the clinical utility of the ≥50 mg/dL (or ≥100-125 nmol/L) cut-off in identifying individuals at higher risk of CAD.

Given the growing incidence of premature CAD and unexplained sudden cardiac deaths, the routine measurement of Lp(a) is becoming increasingly essential. Despite its established role as an independent risk factor, Lp(a) is still not widely incorporated into current cardiovascular prevention guidelines. Our findings show that 30.09% of the North Indian population studied had elevated Lp(a) levels, suggesting that nearly one in three individuals may be at risk for developing complex CAD. This highlights an urgent need to integrate Lp(a) screening into standard risk assessment protocols, which could play a crucial role in early identification and prevention of CAD, particularly in high-risk and younger populations.

This study has several potential limitations. Being retrospective and observational in nature, it is susceptible to selection bias and unmeasured confounding factors that may affect the observed association between Lp(a) levels and SYNTAX score. It was also a single-centre study conducted exclusively in a North Indian population, which may limit the generalizability of the findings to broader populations. Additionally, the absence of a control group restricted our ability to compare Lp(a) levels with individuals without CAD. The use of the SYNTAX score introduces a degree of inter-observer variability, which may influence the assessment of CAD complexity.

## Conclusions

Based on our findings, Lp(a) serves as an independent biomarker for complex CAD. An Lp(a) level exceeding 50 mg/dL was significantly associated with a higher likelihood of anatomically complex CAD, defined by a SYNTAX score >22, independent of traditional cardiovascular risk factors. This threshold demonstrated good diagnostic performance, with a sensitivity of 63.37%, specificity of 75.14%, and an overall accuracy of 71.91%, supporting its clinical relevance in identifying patients at higher risk for complex CAD.
